# The potential population‐based impact of an HPV vaccination intervention in Colorado

**DOI:** 10.1002/cam4.2803

**Published:** 2019-12-23

**Authors:** Jessica R. Cataldi, Marian Håbesland, Amy Anderson‐Mellies, Amanda F. Dempsey, Myles Cockburn

**Affiliations:** ^1^ Adult and Child Consortium for Health Outcomes Research and Delivery Science University of Colorado Anschutz Medical Campus and Children’s Hospital Colorado Aurora CO USA; ^2^ Department of Pediatrics University of Colorado Anschutz Medical Campus Aurora CO USA; ^3^ Colorado School of Public Health Aurora CO USA; ^4^ University of Colorado Cancer Center Aurora CO USA

**Keywords:** cervical cancer, human papillomavirus vaccines, preventive medicine, vaccination

## Abstract

**Background:**

Human papillomavirus (HPV) infection is the most common cause of cervical cancer and can be prevented with vaccination, but HPV vaccination rates remain low. An intervention to improve health care provider communication about vaccination has been shown to increase HPV vaccination rates in an initial trial in Colorado, where about 160 cases of cervical cancer are diagnosed each year.

**Methods:**

Census data were combined with Colorado cancer and immunization registry data to identify clinics in locations that would most benefit from implementation of this intervention to improve HPV vaccination rates. ArcGIS Pro was used to map cervical cancer incidence, immunization rates, population data, and location of clinics participating in practice‐based research networks (PBRNs). Results from the provider communication intervention trial and published estimates of the number needed to vaccinate to prevent a case of cervical cancer were used to predict the number of cervical cancer cases prevented based on increased vaccination due to the intervention.

**Results:**

Ninety‐eight Colorado PBRN clinics were analyzed. For the 10 clinics with the highest predicted number of cervical cancer cases prevented, 5218 additional patients would be vaccinated and 43 cervical cancer cases prevented with implementation of the intervention. If implemented in all 98 clinics, the intervention would lead to 20 490 additional patients vaccinated (range 7‐658/clinic) and 171 cases of cervical cancer prevented (range 0.05‐5.48/clinic).

**Conclusions:**

Geographic data from cancer and immunization registries can inform the dissemination of evidence‐based practices like the provider communication intervention for HPV vaccination to maximize impact on public health.

## Graphical abstract

A model predicting the number of cervical cancer cases prevented with implementation of an evidence‐based intervention to increase HPV vaccination was developed using cancer and immunization registry data and published estimates of the number needed to vaccinate to prevent one case of cervical cancer. Population, cervical cancer incidence, immunization rates, and clinic locations across Colorado were analyzed to identify clinics that would benefit most from the intervention. This analysis demonstrates how spatial data from existing registries can identify clinics with the highest predicted number of cervical cancer cases prevented, thus maximizing the public health impact of an HPV vaccination intervention.

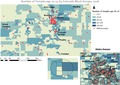

## 1 INTRODUCTION

The incidence of cancers associated with human papillomavirus (HPV) is estimated to be over 43 000 cases in the US per year including cancers of the cervix, vagina, vulva, penis, anus, and a subset of those in the oropharynx.^1^ Cervical cancer represents 27% of all HPV‐related cancers in the US with an average of 12 000 cases diagnosed each year from 2012‐2016.^1^ Over 90% of all cases of cervical cancer can be attributed to HPV, whereas about 70% of oropharyngeal cancers are attributed to HPV.^2^ For this reason, we focused on cervical cancer in Colorado to model the potential impact of dissemination of an intervention to improve HPV vaccination. HPV vaccines, highly effective at preventing HPV infection, have been available since 2006 for females and since 2009 for males. However, in 2018, only 68% of US adolescents aged 13‐17 years had started the vaccination series and only 51% of adolescents had finished it.^3^

Health care professionals’ recommendation for HPV vaccination has a large impact on whether adolescents receive the vaccine,^4^, ^5^ yet health care professionals are often ineffective at HPV vaccine communication.^6^, ^7^, ^8^, ^9^, ^10^ A practice‐based trial in pediatric and family medicine clinics in Colorado demonstrated the effectiveness of an intervention to improve provider communication in increasing adolescent HPV vaccine uptake.^11^ When considering dissemination of this intervention, optimal use of resources would involve targeting the intervention to specific clinics in populations where the need is highest—either in terms of HPV cancer rates, low vaccination rates or a combination of both. In Colorado, approximately 160 cases of cervical cancer are diagnosed every year.^12^ In 2018, 77% of Colorado adolescents had started the HPV vaccination series and 63% had finished.^3^ The objectives of this study were to use population‐based cancer registry and immunization information system data to: (a) determine the likely impact of this provider communication intervention if it was to be conducted at other clinics throughout Colorado and (b) identify those clinics most beneficial to target on the basis of local vaccination rates, distribution of the population eligible for vaccination, and local cervical cancer rates. We predicted the effect of the provider communication intervention in areas surrounding existing Practice‐Based Research Network (PBRN) clinics. PBRN clinics partner with researchers to test and implement public health interventions and could be used to expand the reach of this previously developed intervention.

## 2 METHODS

### 2.1 Previously developed clinic‐based intervention to increase hpv vaccination

The HPV provider communication study used a randomized controlled clinical trial design with covariate‐constrained cluster randomization of 16 practices. Eight practices were included in the intervention group, and each of the practices in the study was concentrated around the most populous metropolitan area of Colorado. The intervention included HPV fact sheets, a parent education website, pictures of diseases caused by HPV, a decision aid for the HPV vaccination, and HPV vaccine communication training for health care professionals. Adolescents in the intervention practices had significantly higher odds of HPV vaccine series initiation (adjusted odds ratio [aOR], 1.46; 95% CI, 1.31‐1.62) and completion (aOR, 1.56; 95% CI, 1.27‐1.92) than those in the control practices (a 9.5—absolute percentage point increase in HPV vaccine series initiation and a 4.4—absolute percentage point increase in HPV vaccine series completion in intervention practices).^11^

### 2.2 Practice‐based research network clinics

**Figure 1:**
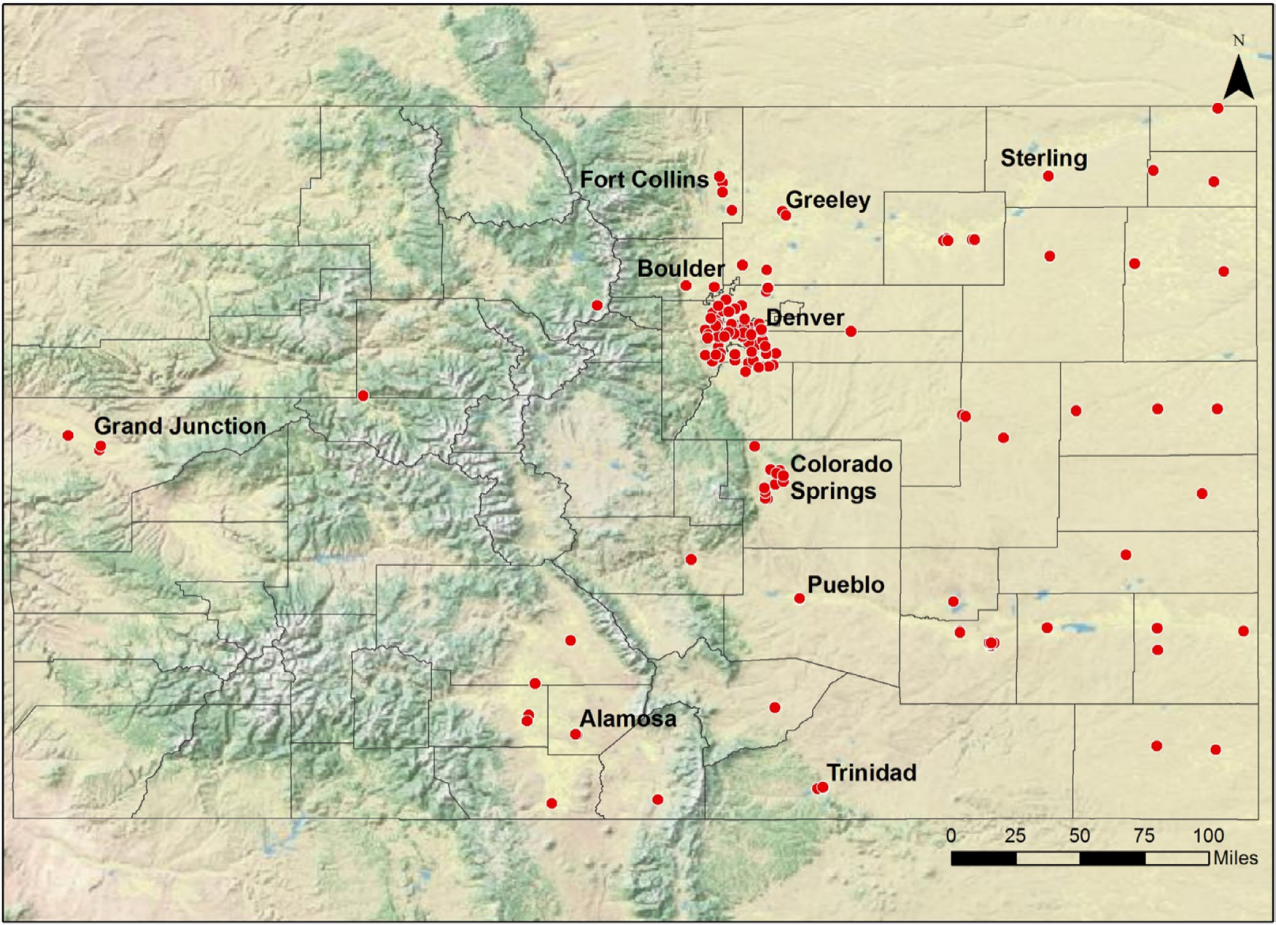
Locations of Colorado PBRN clinics considered for HPV vaccination intervention (N = 174)

There were 174 clinics affiliated with Colorado PBRNs that were considered for this study. These PBRN clinics are situated in both rural and urban areas around Colorado (Figure 1) and include internal medicine, pediatric, and family medicine clinics. Clinics that did not see females between the ages of 9 and 26 (for example senior or geriatric clinics) were excluded (N = 1). Although the initial HPV vaccine provider communication trial was focused on adolescent patients, for this study we chose to include clinics seeing patients aged 9‐26 years (the ages for which HPV vaccination is recommended). PBRN clinics that had been involved in the initial intervention study, either as intervention clinics or controls, were also excluded (N = 15) because they had already been exposed to intervention materials and would not be targeted for future dissemination.

### 2.3 Census data

**Figure 2:**
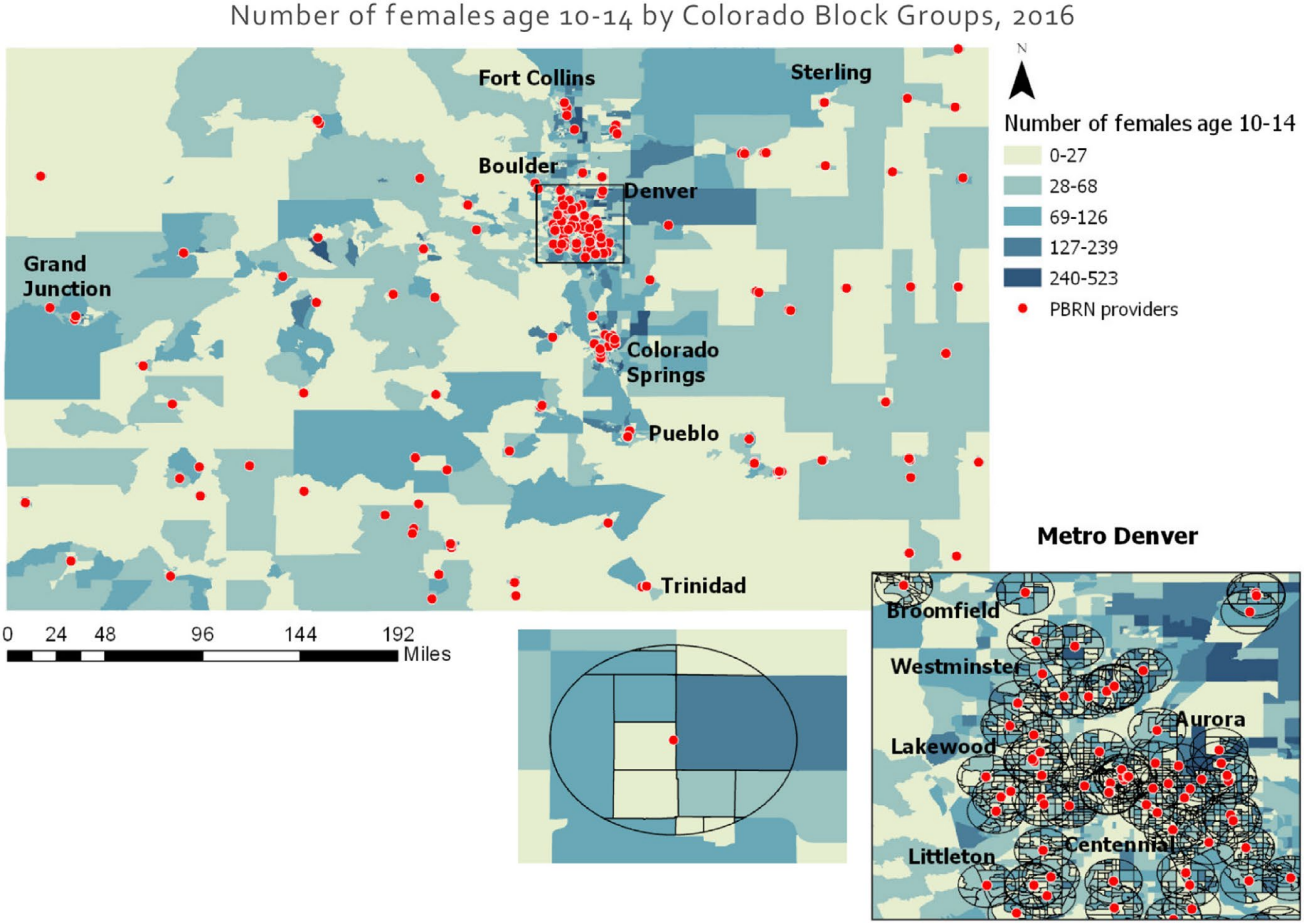
Example of map used to calculate number of females in block groups in 2‐mile buffers around PBRN clinics

Population data from the 2016 American Community Survey 5‐year Estimates^13^ for all Colorado block groups were mapped using ArcGIS Pro 2.1.0 to determine the number of females eligible for vaccination in 2‐mile buffers surrounding the PBRN clinics. Population counts were obtained for females in the 5‐year age grouping (ages 5‐9, 10‐14, 15‐19, and 20‐24) that best encompassed ages for which HPV vaccination is recommended (ages 9 to 26).^14^ Block group population counts were adjusted proportionally to the geographic area of the block group contained within the 2‐mile buffer. This process is represented in Figure 2.

### 2.4 Cervical cancer incidence data

Incidence data for cervical cancer among women under age 65 from 2006 to 2015 in Colorado were collected using the Colorado Central Cancer Registry (CCCR), the state's population‐based cancer registry that includes the geocoded residence of cases at the time of diagnosis. All Colorado residents who are diagnosed with cancer are included in the database, regardless of whether or not they receive their diagnosis in Colorado.^12^ We analyzed cervical cancer data for a 10‐year period to minimize the impact of year‐to‐year variation in incidence and started from the time that the HPV vaccine became available in 2006.

Incidence density maps were created using ArcMap 10.3.1 to show the distribution of cervical cancer cases in Colorado based on the residence location of each case.^12^ These data were intersected with the locations and 2‐mile buffer zones of the PBRN clinics. The number of patients predicted to be vaccinated and the predicted number of cervical cancer cases that would be prevented was calculated for all clinics, however we were unable to calculate the preventable fraction of cervical cancer cases for those clinics without cervical cancer cases in their buffer zone. PBRN clinics that did not have any cervical cancer cases within their 2‐mile buffer zone between 2006 and 2015 (N = 60) were excluded from further analysis. For calculation of the preventable fraction of cervical cancer cases, baseline cumulative age‐adjusted rates of cervical cancer diagnosed among females under age 65 over a 10‐year period (2006‐2015) were calculated for the areas surrounding the remaining PBRN clinics. Rates were calculated using cervical cancer case counts for 2006‐2015 and 2010 Census population data for females 0‐64 years of age for census tracts within the 2‐mile clinic buffer, which were then age‐adjusted to the 2000 US standard population.

### 2.5 HPV vaccination data

Zip‐code level HPV vaccination data from 2017 was obtained from the Colorado Immunization Information System (CIIS), collected by the Colorado Department of Public Health and Environment. These data included females aged 11‐17 years that had received at least one dose of the HPV vaccination. The number of vaccinated females aged 11‐17 years in intersecting zip codes of the 2‐mile buffers was divided by the total number of females age 11‐17 in the zip codes, to create baseline vaccination rates for the clinics. Zip‐code level vaccination rates were used to estimate clinic immunization rates because obtaining clinic‐specific immunization data from all PBRN clinics was not feasible with existing data and privacy infrastructure limitations. A predicted change in vaccination rate post‐intervention was calculated for PBRN clinics using a linear regression model derived from the initial HPV provider communication intervention study, looking at the association between baseline clinic vaccination rates and the change in vaccination rate from pre‐ to post‐intervention.^11^

### 2.6 Determining the number needed to vaccinate

The estimated NNV was based off a literature review, where a systematic review study was used to determine this value.^15^ The systematic review included NNVs of 120, 129 and 324 in three different studies.^16^, ^17^, ^18^ Both 120 and 324 were used in a sensitivity analysis of possible scenarios, and 120 was used for the final analyses presented in this paper.

### 2.7 Preventable fraction

The preventable fraction of cervical cancer cases was calculated for each clinic and was defined as the proportion of cervical cancer that we would expect to prevent in the population at risk by increasing HPV vaccination rates with the provider communication intervention. The number of people that we would expect to vaccinate with the intervention (NV_PI_) was first calculated using the population eligible for vaccination and the baseline and post‐intervention vaccination rates. The cervical cancer cases we would expect to prevent with the intervention was then calculated by dividing the NV_PI_ by the estimated NNV. The expected post‐intervention incidence was calculated using the baseline number of cases, the cases prevented with the intervention and the population at risk. Lastly, the preventable fraction was calculated using the baseline cervical cancer incidence and the expected post‐intervention cervical cancer incidence.

## 3 RESULTS

Ninety‐eight PBRN clinics (Table 1) were included in the final calculations after 75 ineligible clinics were excluded (1 senior clinic, 15 clinics that participated in initial trial, and 60 clinics without incident cervical cancer cases in their 2‐mile buffer zone). The expected increase in HPV vaccination rates varied from 0.3% to 11.2% when applying the linear regression model derived from to initial study^11^ to predict each clinic's post‐intervention vaccination rates.

**Table 1:** Characteristics of practice‐based research network clinics included in full analyses, n = 98 clinics

Characteristic	% (n)
Type of practice	
Pediatric	45% (44)
Family medicine	43% (42)
Internal medicine	6% (6)
Multi‐specialty	6% (6)
Practice location	
Urban	87% (85)
Rural	13% (13)

### 3.1 Predicted impact: 10 clinics with highest predicted number of cervical cancer cases prevented

The 10 clinics with the highest predicted number of cervical cancer cases prevented are summarized in Table 2 including the predicted number of cervical cancer cases prevented based on estimated NNV = 120 and estimated NNV = 324. The results presented in the remainder of this manuscript use estimated NNV = 120. Eight of the 10 clinics in Table 2 were located in one metropolitan area, while the remaining two clinics were in two other distinct locations. For these 10 clinics, the total number of additional patients predicted to be vaccinated if the intervention was implemented was 5218, and the total predicted number of cervical cancer cases prevented was 43.

**Table 2:** Projected impact of the provider communication for HPV vaccination intervention for the 10 PBRN clinics with the highest predicted numbers of cervical cancer cases prevented

PBRN clinic	Vaccination rates (%)^a^	Vaccinated with intervention (n)	Baseline cervical cancer cases (n)	Preventable fraction of cervical cancer cases^b^ if NNV = 324	Preventable fraction of cervical cancer cases if NNV = 120
Clinic 1	Pre‐intervention: 42.0	658	4.86	41.8%	100.0%^c^
	Post‐intervention: 50.3				
Clinic 2	Pre‐intervention: 44.5	647	6.68	29.9%	80.7%
	Post‐intervention: 52.0				
Clinic 3	Pre‐intervention: 42.3	629	4.34	44.7%	100.0%^c^
	Post‐intervention: 50.5				
Clinic 4	Pre‐intervention: 41.6	627	8.34	23.2%	62.7%
	Post‐intervention: 50.0				
Clinic 5	Pre‐intervention: 42.9	553	3.70	46.1%	100.0%^c^
	Post‐intervention: 50.9				
Clinic 6	Pre‐intervention: 44.5	468	3.46	41.7%	100.0%^c^
	Post‐intervention: 52.0				
Clinic 7	Pre‐intervention: 45.9	447	8.10	17.0%	46.0%
	Post‐intervention: 53.0				
Clinic 8	Pre‐intervention: 47.6	409	6.28	20.1%	54.3%
	Post‐intervention: 54.2				
Clinic 9	Pre‐intervention: 58.4	396	9.51	12.9%	34.7%
	Post‐intervention: 61.7				
Clinic 10	Pre‐intervention: 55.5	384	6.32	18.8%	50.7%
	Post‐intervention: 59.7				
Total for clinics 1‐10		5218	61.59	26.1%	70.6%

Abbreviations: PBRN, practice‐based research network; NNV, number needed to vaccinate to prevent one case of cervical cancer.

a Expected post‐intervention HPV vaccination rate calculated using linear model based off initial trial of the provider communication intervention.

b Preventable fraction of cervical cancer cases calculated by dividing number of cases predicted to be prevented by number of cases occurring in 2‐mile spatial buffer zone surrounding clinic. Number of cases predicted to be prevented calculated by dividing the number of people predicted to be vaccinated with intervention by the number needed to vaccinate to prevent one case of cervical cancer

c The estimated preventable fraction of cervical cancer cases exceeded 100% because the estimate of cases prevented exceeded the number of cases occurring in the population in the 2‐mile buffer surrounding the clinic

### 3.2 Predicted impact: all 98 PBRN clinics analyzed

**Figure 3:**
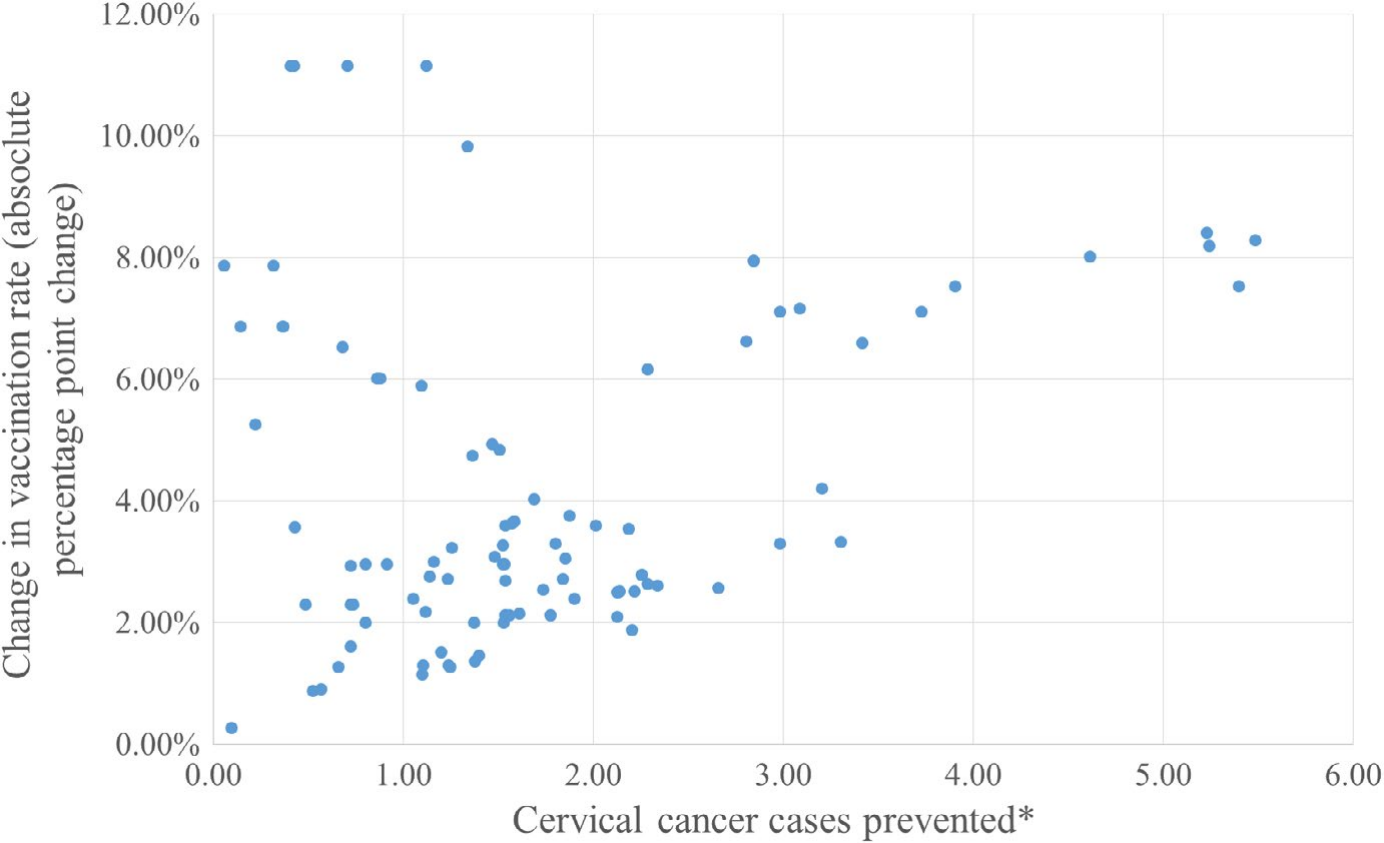
Predicted increase in vaccination rate and number of cervical cancer cases prevented by PBRN clinic (n =98). ^*^Based on number needed to vaccinate to prevent one case of cervical cancer (NNV) = 120

The number of additional individuals predicted to be vaccinated with implementation of the intervention ranged from 7 to 658 people across the 98 clinics analyzed. The predicted number of cervical cancer cases prevented ranged from 0.05 to 5.48 (Figure 3) and the preventable fraction ranged from 1.7% to >100.0%. The estimated preventable fraction of cervical cancer cases exceeded 100% for some clinics because the estimated number of cases prevented (based on estimated increase in vaccination rates) exceeded the number of cases of cervical cancer occurring in the population in the buffer surrounding the clinic. The total number of additional patients predicted to be vaccinated if all 98 clinics were targeted with the intervention was 20,490, and the total predicted number of cervical cancer cases prevented was 171.

## 4 CONCLUSIONS

This study identified clinics in Colorado that had the highest predicted number of cervical cancer cases prevented and would therefore be most beneficial to target with a HPV vaccine provider communication intervention. The methods used demonstrate how population‐based data can be used to target dissemination of an intervention. Several PBRN clinics located in one metropolitan area were found to reside in populations with the highest number of preventable cervical cancer cases, in part because these clinics surrounding populations have lower baseline vaccination rates and a higher predicted change in vaccination rates from baseline to post‐intervention. In addition, the 2‐mile buffer zones around these clinics encompass a large population at risk, contributing to high numbers of patients predicted to be vaccinated due to the intervention.

HPV vaccination is known to be very effective at preventing cervical cancer but is currently underutilized. An effective clinic‐level intervention to improve provider communication about HPV vaccine has been implemented in family medicine and pediatric clinics in the Colorado's Front Range area in an initial trial. Now the intervention is ready to be disseminated to other Colorado clinics and extended to improving “catch up” HPV vaccination among those 18‐26 years of age. In the current analysis, we identified locations of PBRN clinics that could help prevent the highest number of cervical cancers by increasing HPV vaccination uptake. While the number of clinics that could be targeted may be determined by available resources and funding, identifying those clinics with the highest predicted number of cervical cancer cases prevented (Table 2) provides a data‐driven method of prioritizing the vaccination intervention.

The predicted number of cases of cervical cancer prevented by implementing the HPV vaccine provider communication intervention may be underestimated by our analyses. One challenge to interpretation of the predicted number of cervical cancer cases prevented is the use of different time horizons for cervical cancer incidence and modeling the number needed to vaccinate to prevent a case of cervical cancer. Studies that model the number needed to vaccinate with HPV vaccine examine the reduction in lifetime risk of HPV‐related outcomes such as cervical cancer. So, for a number needed to vaccinate of 120, vaccinating 120 people would prevent one case of cervical cancer over a lifetime. The estimate of 171 cases of cervical cancer prevented by implementing the HPV provider communication intervention in all PBRN clinics is not directly comparable to the population‐based incidence of cervical cancer in Colorado, which is about 160 cases per year, because these 171 cases are prevented over the lifetimes of those vaccinated. In addition, the predicted increase in HPV vaccination rates was based on a static estimate of the population at risk in the 2‐mile buffer zones surrounding each PBRN clinic and does not account for people aging into (and out of) the 9‐26 year age range of eligibility for routine HPV vaccination. If increased vaccination rates are sustained after the intervention (which remains to be studied), future age cohorts would also benefit from increased vaccination rates and contribute to the predicted number of cervical cancer cases prevented in the future.

The methods presented in this study illustrate one way to use cancer registry and immunization registry data to target vaccination interventions for primary prevention of HPV‐related cancer. Data from of cancer registries in the US and around the world have contributed tremendously to the understanding of epidemiologic trends and helped to identify factors associated with cancer diagnosis and outcomes. Data from immunization registries are often used to identify areas or populations with low vaccination rates or to assess changes in vaccination rates after interventions at the practice, public health, or policy level. By combining geographic information from both the cancer registry and the immunization registry, we used existing data sources to identify locations where high HPV‐related cancer incidence and low HPV vaccination rates overlapped. Each state in the US has its own central cancer registry^19^ and most also have state‐level immunization registries,^20^ thus making this approach for targeting HPV vaccination interventions replicable outside of Colorado.

Spatial analysis of vaccination and cervical cancer rates can inform an approach focused on the population at risk, however, identifying the population at risk is only a part of ensuring optimal dissemination of HPV vaccination interventions. Additional study is needed to identify other factors that may predict the success of the interventions like the HPV provider communication intervention in various practice settings such as provider type, practice size, and organizational factors. We are currently using qualitative methods to conduct pre‐implementation assessments among providers from PBRN clinics in at‐risk locations.

### 4.1 Strengths and limitations

There were several limitations in this study that could have an impact on the results or the generalizability of the study. First, it is difficult to predict how far individuals are willing to travel to seek primary care. A 2‐mile buffer was drawn around PBRN clinics for these analyses which may not reflect the true population of patients seen at each clinic, particularly in rural areas where people often travel long distances to seek care. For the PBRN locations in urban areas, a 2‐mile buffer zone helped to decrease overlap in clinic catchment areas. Because we do not have clinic‐level data about the location of residence for each clinic's actual patient population, we used the same size buffer for every clinic. We assumed an even distribution of populations within block groups, however if population density varies within block groups, then the estimated population at risk within the 2‐mile buffer zones may not be accurate. Some clinic estimates for the preventable fraction of cervical cancer cases exceeded 100%, implying that we did not have an accurate estimate of the population at risk surrounding those clinics. We did not know the true population at risk surrounding each clinic, nor could we without conducting a detailed survey of each clinic's patient population. Our approach still identifies those clinics most likely to benefit from receiving the intervention, even if the absolute value of the preventable fraction of cervical cancer cases is over‐estimated. An additional limitation of our approach is that we did not predict the impact of adolescents changing their location of residence after vaccination, which could have varying effects on the number of cervical cancer cases prevented in each location.

Zip code level vaccination rates may not reflect the population truly attending a specific clinic in that location. Clinics might draw their patients from farther afield than their local zip code or from only one part of their local zip code that might have a different vaccination rate than the zip code average represents. Use of zip code level vaccination data is imperfect but allows for use of population‐level data from an existing database (CIIS) rather than collecting data separately for each clinic. The estimated NNV for HPV vaccine to prevent one case of cervical cancer is reported across a broad range in the published literature (120‐324) and does not account for differences in vaccine effectiveness based on age at vaccination. The variability in this value may have impacted our analyses by biasing the results toward finding a higher predicted number of cervical cancer cases prevented because we chose the lower estimated NNV, however this bias would be unlikely to change the relative distribution of cases prevented across the geographic areas analyzed. In addition, we used rates of HPV vaccine initiation rather than completion for our analyses. Published estimates of NNV to prevent cervical cancer are based upon HPV vaccine completion; however, emerging data may support the effectiveness of single‐dose HPV vaccination,^21^, ^22^ thus making it difficult to determine whether the predicted number of cervical cancer cases prevented would change by using completion rather than initiation rates.

The analysis presented here looked at predicted impact of an intervention to increase HPV vaccination rates among populations ages 9‐26, however the initial HPV provider communication intervention was not tested beyond the adolescent period and the baseline vaccination rates examined were for adolescents 11‐17 years. It is unknown whether the gains of the HPV provider communication intervention would also be seen among young adults. This study focused on cervical cancer and HPV vaccination among females to demonstrate the modeling methods. In future work, we will incorporate vaccination rates and HPV‐related cancers among males. Because the referenced intervention was based at the clinic level without specific focus on patient‐level socioeconomic characteristics, we did not assess for such factors in this geospatial model. We plan to assess socioeconomic factors using census data in future work. Generalizability of our findings may be limited as PBRN clinics may differ from primary care clinics that are not part of a research network.

HPV vaccination is a powerful and underutilized tool for preventing cervical cancer. We present an example of how geographic data from cancer and immunization registries can be used to identify clinics with the highest predicted number of cervical cancer cases prevented if an intervention was to be implemented. These methods should be applied in planning the dissemination and implementation of interventions like the provider communication intervention for HPV vaccination in order to maximize the predicted impact on public health.

## DISCLOSURES

Amanda Dempsey serves on advisory boards for Merck, Pfizer and Sanofi. She did not receive research support from these companies. The other authors have no potential conflicts of interest.

## AUTHOR CONTRIBUTIONS

Jessica Cataldi: Data curation, project administration, writing—original draft, and writing—review and editing. Marian Håbesland: conceptualization, data curation, formal analysis, visualization, writing—original draft, and writing—review and editing. Amy Anderson‐Mellies: data curation, formal analysis, investigation, methodology, software, validation, visualization, writing—original draft, and writing—review and editing. Amanda Dempsey: Conceptualization, methodology, writing—review and editing. Myles Cockburn: Conceptualization, methodology, resources, supervision, writing—review and editing. In addition, all authors have reviewed the submitted manuscript, provided final approval of this version, and agree to be accountable for all aspects of the work.

## Data Availability

The data that support the findings of this study are available on request from the corresponding author. The data are not publicly available due to privacy or ethical restrictions.
